# MTDH promotes glioma invasion through regulating miR-130b-ceRNAs

**DOI:** 10.18632/oncotarget.14717

**Published:** 2017-01-18

**Authors:** Liping Tong, Ming Chu, Bingqing Yan, Weiyi Zhao, Shuang Liu, Wei Wei, Huihuang Lou, Shengkun Zhang, Shuai Ma, Juan Xu, Lanlan Wei

**Affiliations:** ^1^ Wu Lien-Teh institute, Department of Microbiology, Harbin Medical University, The Heilongjiang Key Laboratory of Immunity and Infection, Pathogen Biology, Harbin 150081, China; ^2^ Department of Neurosurgery, The First Affiliated Hospital, Harbin Medical University, Harbin 150001, China; ^3^ Jiamusi University, Jiamusi 154002, China; ^4^ College of Bioinformatics Science and Technology and Bio-Pharmaceutical Key Laboratory of Heilongjiang Province, Harbin Medical University, Harbin 150081, China

**Keywords:** glioma, MTDH, EMT, miR-130b, ceRNA

## Abstract

Cell invasion is crucial for high mortality and recurrence rate in glioma. Epithelial-mesenchymal transition (EMT) is an important step in cancer invasion. Metadherin (MTDH) contributes to EMT in several cancers, but the role and mechanism of MTDH in EMT-like process of glioma remain unknown. Here we demonstrate that MTDH was overexpressed in glioma tissues and cells and induced EMT-like change and invasion of glioma cells. Interestingly, MTDH could modulate the expression of a group of glioma-related miRNAs. In particular, MTDH upregulated miR-130b transcription via acting as a coactivator of NF-kB. MiR-130b promoted EMT-like change and invasion of glioma cells through targeting multiple EMT-related genes, including PTEN, PPP2CA and SMAD7. In addition, PTEN acted as the competing endogenous RNA (ceRNA) to affect PPP2CA and SMAD7 expression, and inhibited EMT-like change in glioma cells. Furthermore, miR-130b mediated EMT-like change induced by MTDH, and MTDH inhibited the expression levels of PTEN, PPP2CA and SMAD7. Taken together, we reveal a novel mechanism that MTDH induces EMT-like change and invasion of glioma via the regulation of miR-130b-ceRNAs, providing the first direct link between MTDH and miRNAs in cancer cells.

## INTRODUCTION

Glioma is the most common and lethal type of primary brain tumor. Tumor cells highly infiltrate and invade into surrounding normal brain tissues, largely accounting for malignant progression and recurrence of glioma, especially for glioblastoma multiforme (GBM) [1]. Currently, there are still no efficient therapeutic approaches for glioma. Epithelial-mesenchymal transition (EMT), a process in which epithelial properties are impaired while mesenchymal properties are enhanced, is a critical step in cancer invasion and metastasis [2]. Thus, exploring the mechanism of EMT-like change in glioma invasion and metastasis will be important to develop effective strategies for glioma treatment.

Metadherin (*MTDH*), also known as astrocyte-elevated gene-1 (*AEG-1*) and *LYRIC*, is an important oncogene that plays a crucial role in the initiation and progression of most malignant tumors [3]. It has been documented that MTDH could modulate gene expression by acting as a cofactor for transcription factors including NF-κB, AP-1, c-MYC and LEF-1 [4]. Our previous study showed that MTDH was highly expressed in glioma and could interact with NF-κB component p65 to promote staphylococcal nuclease domain containing 1 (SND1) expression [5]. MTDH has been shown to induce EMT in several tumors and promote tumor invasion [6–8].

EMT is triggered by several layers of regulation network, including transcriptional, non-coding RNA, alternative splicing, translational and posttranslational regulation [2]. MicroRNA (miRNA), a type of small non-coding RNA, regulates gene expression by binding to miRNA response elements (MREs) on target genes [9]. Recent studies suggest that competing endogenous RNAs (ceRNAs) contain the same MREs and cross-regulate each other through sequestering miRNAs to form a complex regulatory network [10]. It has been reported that the silencing function of miRNA is modulated by MTDH which interacts with SND1 in RNA-induced silencing complex (RISC) to facilitate RISC activity [11]. However, the roles of MTDH in regulating miRNAs expression and miRNA-ceRNAs remain elusive.

We hypothesized that MTDH might drive EMT-like change through regulating miRNA-ceRNAs network to promote glioma invasion. In this study, we provided the first evidence that MTDH regulated glioma-related miR-130b expression. Furthermore, miR-130b promoted glioma EMT-like change by targeting PTEN, PPP2CA and SMAD7, and PTEN acted as the ceRNA for PPP2CA and SMAD7 to inhibit EMT-like change of glioma cells.

## RESULTS

### MTDH promotes EMT-like change and invasion of glioma cells

**Figure 1:**
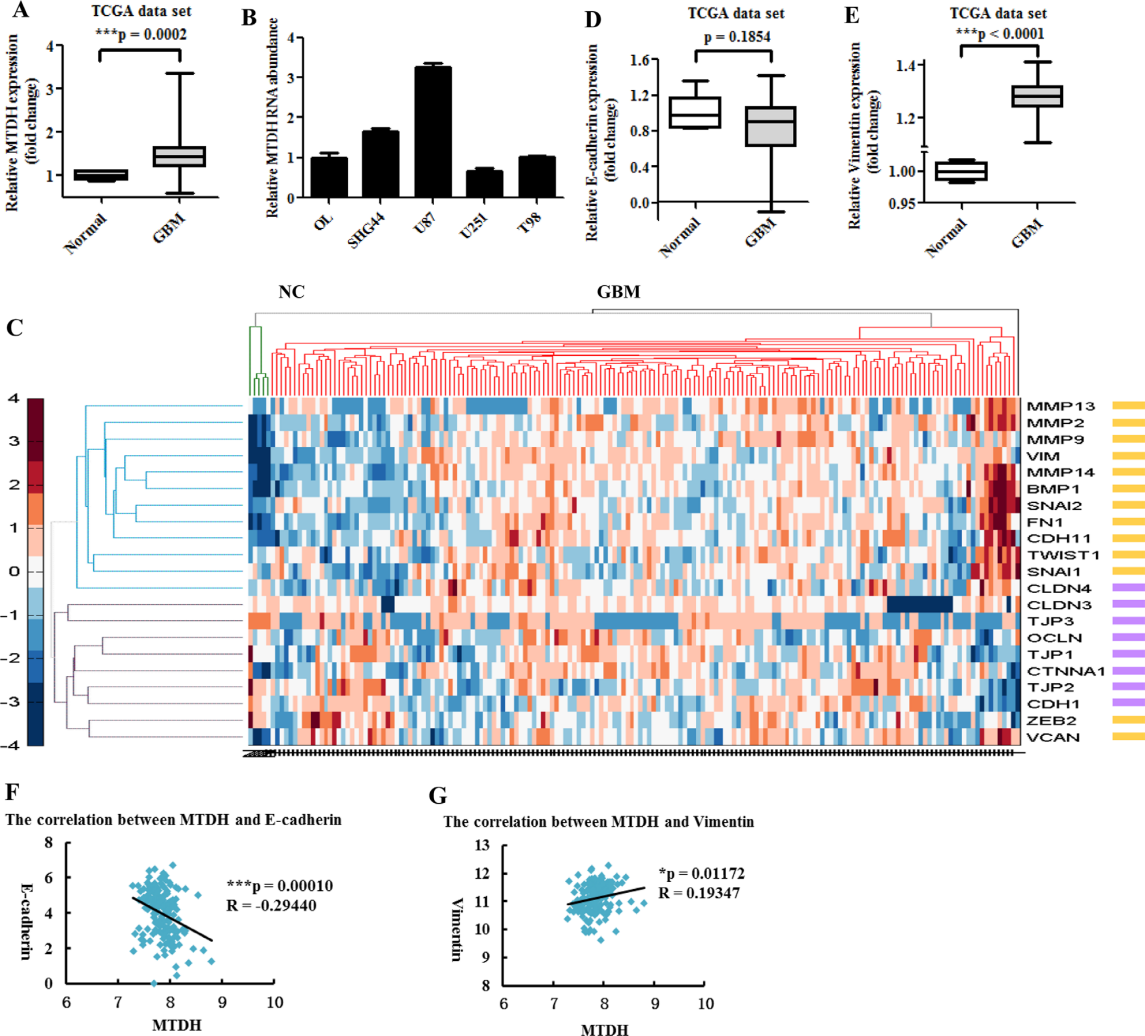
MTDH and mesenchymal markers are highly expressed in GBM (**A**) Box plot of MTDH expression in GBM (*n* = 473) and normal brain (*n* = 10) samples available from TCGA database. (**B**) Quantitative RT-PCR analysis of MTDH expression in various glioma cell lines and normal glia OL cells. (**C**) Unsupervised hierarchical clustering analysis of epithelial and mesenchymal genes in GBM and normal brain samples based on TCGA dataset. Green line represents normal brain and red line represents GBM. Purple cube denotes epithelial gene, while yellow cube represents mesenchymal gene or EMT-TF. CDH1: E-cadherin gene. VIM: Vimentin gene. (**D**, **E**) Analysis of E-cadherin and Vimentin expression in GBM and normal samples from TCGA. (**F**, **G**) Analysis of the correlation between MTDH and E-cadherin or Vimentin in TCGA GBM samples.

Our previous study showed that MTDH expression was high in glioma tissues and cells and associated with glioma grades [5]. We further confirmed high *MTDH* expression in large numbers of GBM tissue samples from The Cancer Genome Atlas (TCGA) database and in various glioma cell lines (Figure 1A and 1B). Next, we examined the expression of EMT-related genes in GBM through analyzing TCGA dataset. Unsupervised hierarchical clustering was performed to analyze the expression of epithelial and mesenchymal genes including EMT-transcription factors (EMT-TFs). We found that GBM had higher expression of almost all mesenchymal genes and EMT-TFs but lower expression of epithelial genes compared with normal brain tissues (Figure 1C). In addition, mesenchymal marker viment expression was significantly increased in GBM, while epithelial marker E-cadherin expression was reduced (Figure 1D and 1E). Furthermore, we analyzed the correlation between MTDH and EMT markers and found that MTDH expression was negatively correlated with E-cadherin but positively correlated with Vimentin (Figure 1F and 1G). These data suggest that MTDH is involved in glioma EMT-like process.

**Figure 2:**
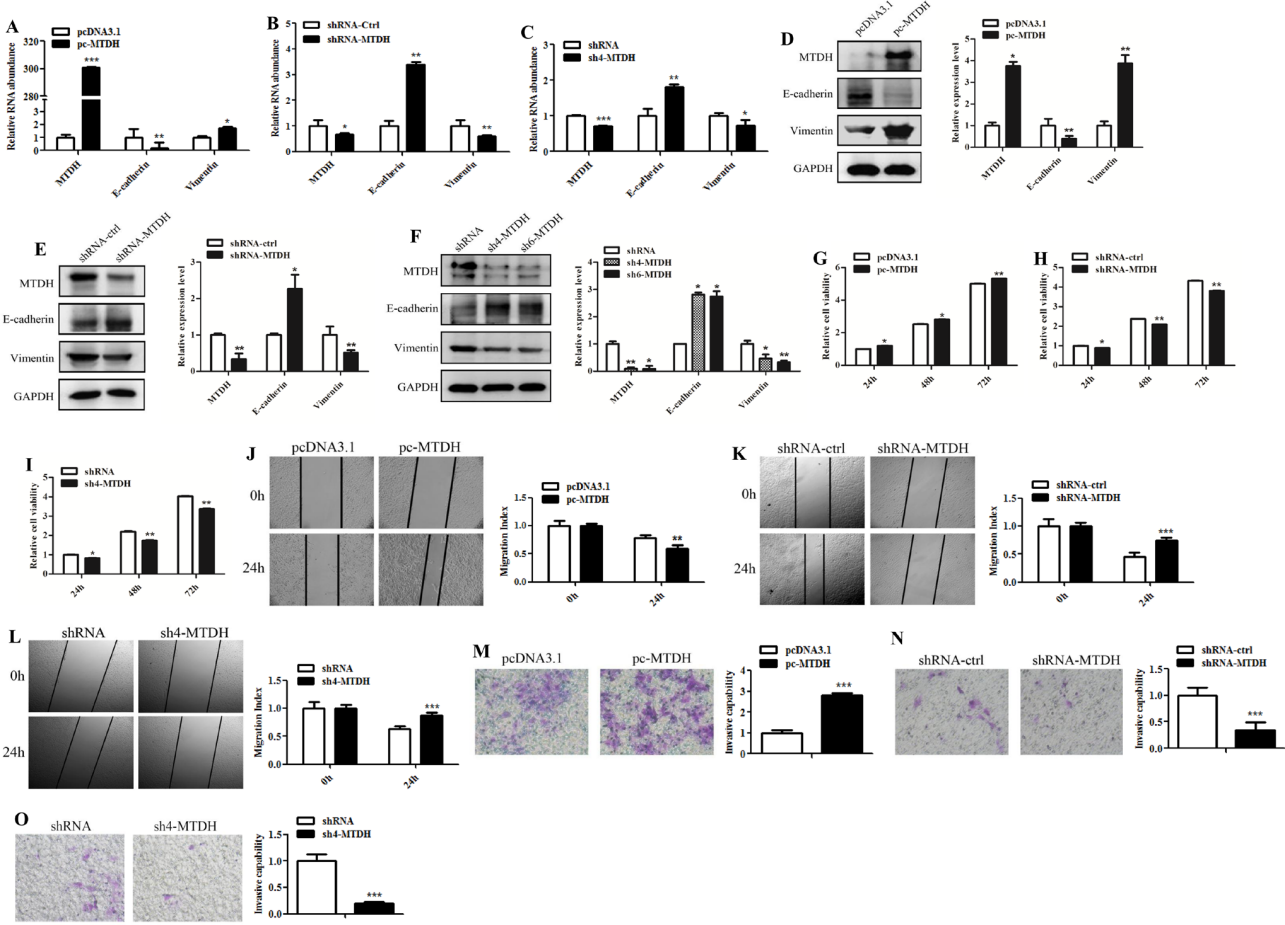
MTDH promotes glioma cells EMT-like change and invasion (**A**–**C**) At mRNA level, the expression changes of EMT-related markers were analyzed by qRT-PCR in glioma cells with overexpression or knockdown of MTDH. (**D**–**F**) Western blot analysis of the expression of EMT-related markers in glioma cells with overexpression or knockdown of MTDH. GAPDH was used as loading control. Densitometric analysis was conducted using IPP softerware. (**G**–**I**) Cell viability was analyzed by MTT assay in glioma cells with MTDH overexpression or knockdown. (**J**–**L**) Cell migration was examined by wound-healing assay in glioma U87 cells with overexpression or knockdown of MTDH. The wound was photographed at 0 h and 24 h after scratching. The area between cells was calculated and normalized to the original area. (**M**–**O**) Cell invasive ability was monitored by transwell matrigel assay in glioma cells with overexpression or knockdown of MTDH. The invaded cells were counted and shown in the histograms. Data represent mean ± SEM of three replicates. *P < 0.05; **P < 0.01; ***P < 0.001.

To investigate whether MTDH regulates EMT-like process to drive glioma invasion, we modulated MTDH expression levels in GBM U87 cells. Quantitative reverse transcription-polymerase chain reaction (qRT-PCR) and Western blot analy showed that E-cadherin expression was decreased but Vimentin expression was increased at mRNA and protein levels in U87 cells after transfection with MTDH overexpression vector pc-MTDH (Figure 2A and 2D). Conversely, E-cadherin expression was enhanced but Vimentin expression was inhibited in U87 cells after transfection with shRNA-MTDH/sh4-MTDH (Figure 2B–2C and 2E–2F). These results indicate that MTDH promotes EMT-like process in glioma cells. Next, we performed wound-healing and transwell matrigel assays to measure the effects of MTDH on the migratory and invasive abilities of U87 cells. MTDH overexpression increased glioma cells viability (Figure 2G), while MTDH knockdown led to the opposite result (Figure 2H–2I). The effect was slight at 24 h, but more obvious 24 h later (Figure 2G–2I). We found that MTDH overexpression enhanced GBM cells migration (Figure 2J) and invasion (Figure 2M). In contrast, MTDH knockdown inhibited GBM cells migration (Figure 2K and 2L) and invasion (Figure 2N and 2O). Collectively, these results indicate that MTDH promotes EMT-like process and invasion of glioma cells.

### MTDH modulates miR-130b expression by acting as a coactivator of NF-κB

**Figure 3:**
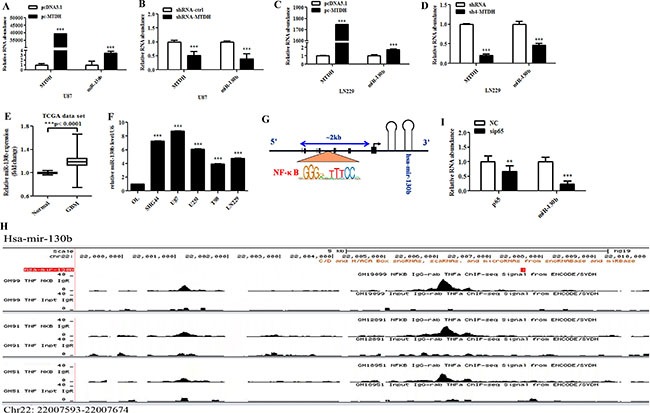
MTDH modulates miR-130b expression by acting as NF-κB coactivator (**A**–**D**) Quantitative RT-PCR analysis of miR-130b expression in U87 and LN229 cells with MTDH overexpression or knockdown. (**E**) Box plot showing the expression level of miR-130b in glioma (*n* = 571) and normal brain (*n* = 10) tissues using TCGA dataset. (**F**) Analysis of miR-130b in normal glia OL cells and various glioma cells. (**G**) The prediction of transcription factor for miR-130b. (**H**) NF-κB subunit p65 peaks were located upstream of the mir-130b gene based on ChIP-seq analysis in human lymphoblastoid cell lines. Data were from ENCODE database, and NF-KB antibody used in IP targeted p65. Red arrow represented the location of mir-130b gene (chr22: 220007593-22007674). (**I**) Analysis of miR-130b expression in glioma cells after transfection with either scramble control or sip65. Data represent mean ± SEM of three replicates.

We next determined whether MTDH could regulate the expression of miRNAs. To search for glioma-associated miRNAs, we analyzed miRNAs expression profiles from TCGA database and focused on a set of miRNAs with different expression among GBM and normal brain tissues (Table 1). We screened these miRNAs based on bioinformatics analysis and microarray data (Supplementary Table S1). Next, we experimentally explored and searched the miRNAs regulated by MTDH. We identified some miRNAs that could be regulated by MTDH, among which miR-130b was significantly regulated (Supplementary Figure S1). In U87 and LN229 cells, we confirmed that miR-130b expression was increased after MTDH overexpression but decreased after MTDH knockdown (Figure 3A–3D). Furthermore, miR-130b was highly expressed in GBM tissues (Figure 3E), and in glioma cell lines compared to glial OL cells (Figure 3F).

**Table 1:** Upregulated miRNAs in GBM compared to normal brain based on TCGA database

Rank	MiRNA	p _t test	FDR	Fold change
1	hsa-miR-21	3.80E-17	7.25E-16	1.5195
2	hsa-miR-27a	2.09E-19	5.58E-18	1.3558
3	hsa-miR-210	4.44E-25	3.07E-23	1.3343
4	hsa-miR-10b	3.33E-17	6.58E-16	1.3289
5	hsa-miR-155	4.06E-28	3.61E-26	1.3139
6	hsa-miR-23a	5.44E-10	2.91E-09	1.2832
7	hsa-miR-25	3.39E-08	1.40E-07	1.2385
8	hsa-miR-106b	4.38E-13	4.33E-12	1.2143
9	hsa-miR-130b	1.36E-20	4.55E-19	1.2122
10	hsa-miR-93	6.69E-22	2.98E-20	1.2062
11	hsa-miR-92b	2.41E-19	6.14E-18	1.2004
12	hsa-miR-196b	9.72E-18	2.08E-16	1.1938
13	hsa-miR-15b	4.15E-08	1.69E-07	1.1816
14	hsa-miR-370	0.002579	0.005577	1.1784
15	hsa-miR-15a	3.86E-13	3.96E-12	1.1753

We wondered how MTDH regulates miR-130b expression. Notably, we identified several NF-κB binding sites upstream of mir-130b gene (Figure 3G). Based on ChIP-seq analysis of genome wide distribution of NF-κB subunits binding regions in human lymphoblastoid cell lines, we found that multiple peaks of NF-κB subunit p65 were located at the upstream of mir-130b gene (chr22: 220007593-22007674) (Figure 3H), indicating that NF-κB regulates the transcription of mir-130b gene. To determine whether NF-κB subunit p65 is involved in modulating miR-130b expression, siRNA-p65 (sip65) and control siRNA were transfected into U87 cells, and p65 knockdown led to the decreased abundance of miR-130b (Figure 3I).

It has been documented that MTDH functions as a coactivator of NF-κB, facilitating the translocation of NF-κB into the nucleus and interacting with the p65 subunit of NF-κB to enhance downstream gene expression [12]. Moreover, our previous study showed that MTDH interacted with p65 in the nucleus and promoted SND1 expression in glioma cells [5]. Thus, MTDH might drive miR-130b expression by acting as the transcriptional coactivator of NF-κB.

### MiR-130b induces EMT-like process and invasion of glioma cells

**Figure 4:**
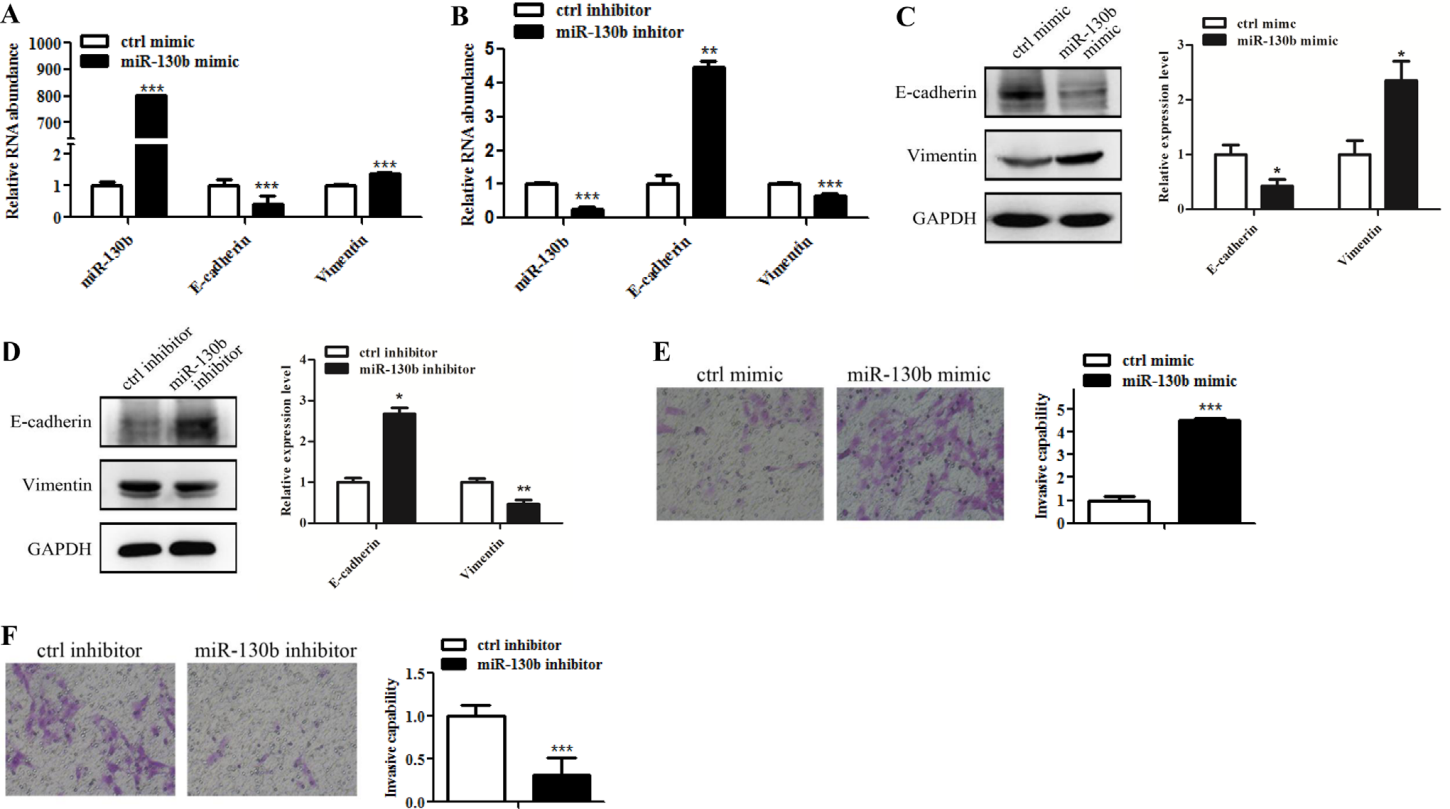
MiR-130b induces EMT-like change and invasion of glioma cells (**A**, **B**) qRT-PCR analysis of E-cadherin and Vimentin expression in glioma cells treated with miR-130b mimic or inhibitor. (**C**, **D**) Western blot analysis of E-cadherin and Vimentin expression levels in glioma cells treated with miR-130b mimic or inhibitor. Relative protein expression levels were analyzed by densitometry. (**E**, **F**) Represent images of transwell invasion assay in glioma cells treated with miR-130b mimic or inhibitor. Data represent mean ± SEM of three replicates.

To understand the functional rol of miR-130b in glioma cells EMT-like process and invasion, we employed miR-130b mimic (or mimic control) and miR-130b inhibitor (or inhibitor control) to modulate miR-130b levels in U87 cells. Quantitative RT-PCR and Western blot analyses showed that miR-130b overexpression led to reduced E-cadherin expression but increased Vimentin expression (Figure 4A and 4C), whereas miR-130b knockdown led to the opposite results (Figure 4B and 4D). Furthermore, wound-healing and transwell matrigel assays showed that miR-130b upregulation increased glioma cells migration and invasion (Figure 4E and Supplementary Figure S2), while miR-130b knockdown suppressed glioma cells invasion (Figure 4F). These data suggest that miR-130b could induce EMT-like process and facilitate glioma invasion.

### MiR-130b target PTEN mediates cross-talk with its ceRNAs and inhibits EMT-like process in glioma cells

**Figure 5:**
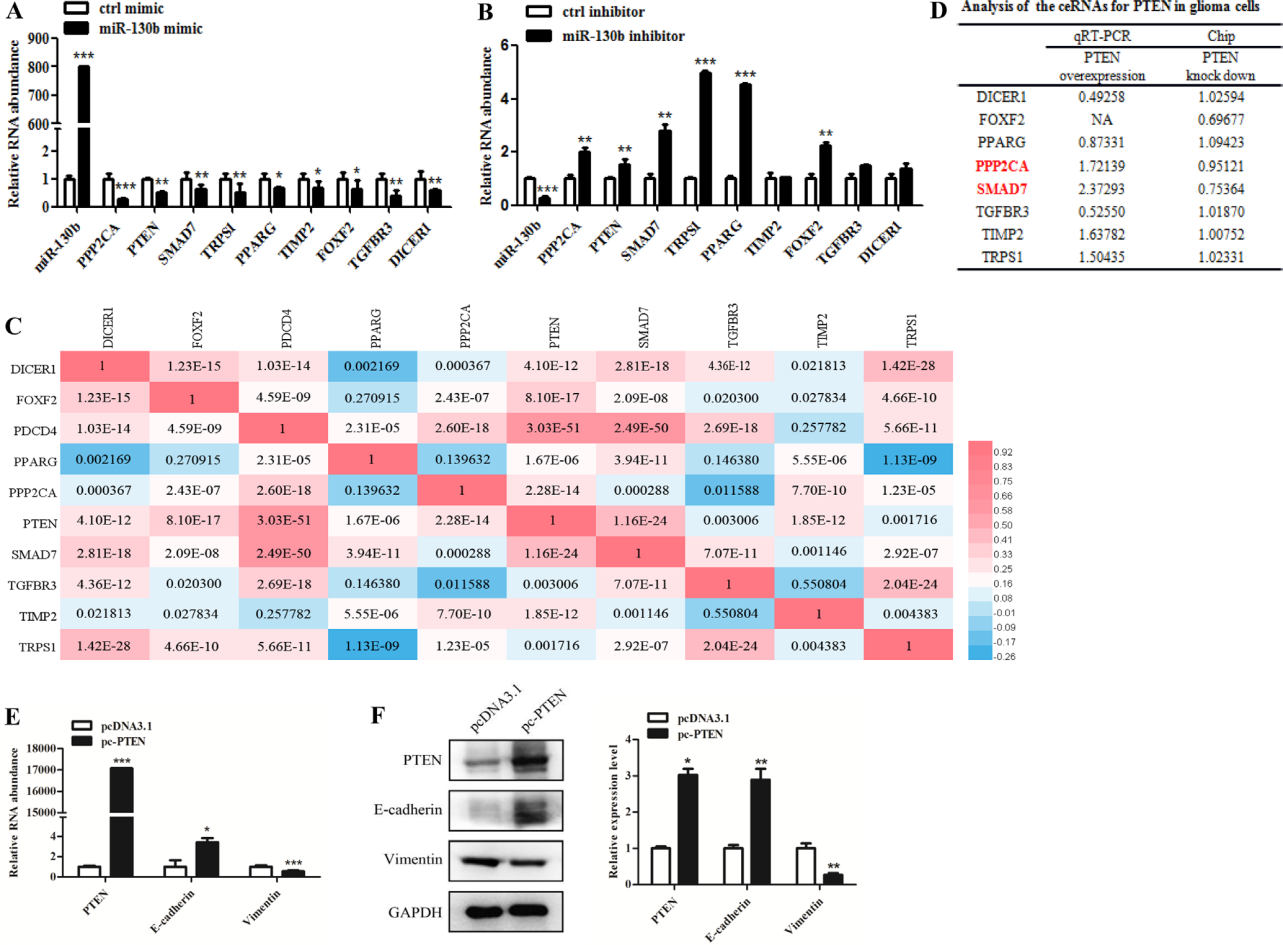
MiR-130b target PTEN mediates cross-talk with its ceRNAs and inhibits EMT-like change in glioma cells (**A**, **B**) qRT-PCR analysis of the expression levels of candidate miR-130b targets in glioma cells with miR-130b overexpression or knockdown. (**C**) The correlations of the expression of miR-130b targets in glioma based on TCGA database. Each colored rectangle illustrated the correlation between two genes. The values represented *p* values for pearson-correlation analyses. (**D**) Quantitative RT-PCR and microarray assays of the expression levels of candidate miR-130b targets in glioma cells with PTEN overexpression or knockdown. (**E**, **F**) The mRNA and protein expression levels of EMT-related markers in glioma cells with PTEN overexpression.

We went on to identify miR-130b targets that mediate the effects of miR-130b on EMT-like process. Based on miRNA target prediction analysis and TCGA miRNA-mRNA expression profile analysis, we selected several candidate miR-130b targets related to EMT-like process (Supplementary Table S2). MiR-130b gain and loss of function experiments were carried out to verify the effects of miR-130b on the candidate targets. We found that PTEN, PPP2CA, SMAD7, TRPS1, PPARG, and FOXF2 expressions were decreased after miR-130b overexpression (Figure 5A), but were increased after miR-130b knockdown (Figure 5B).

Based on Pearson-correlation analysis of miR-130b targets expression in glioma (TCGA database), some targets were observed to be positively related to others and they might become potential ceRNAs. Notably, both PTEN and SMAD7 were correlated with all other targets, and might play significant roles to mediate the effects of miR-130b (Figure 5C and Supplementary Table S3). PTEN is a well-known tumor suppressor and PTEN mutations are usually associated with increased invasion of glioma [13]. Therefore, we focused on PTEN to investigate PTEN-associated ceRNAs and explore their roles in glioma. Microarray (Chip) and qRT-PCR assays showed that PPP2CA and SMAD7 expression levels were increased after PTEN overexpression but decreased after PTEN knockdown (Figure 5D), indicating that PPP2CA and SMAD7 might be the ceRNAs of PTEN to compete miR-130b.

Furthermore, we investigated the role of PTEN in EMT-like process of glioma cells. It was observed that PTEN overexpression led to increased E-cadherin expression but decreased Vimentin expression at mRNA and protein levels in glioma cells (Figure 5E and 5F). These data indicate that miR-130b may regulate EMT-like process through ceRNAs cross-talk between PTEN and PPP2CA/SMAD7.

### MiR-130b-ceRNAs mediates EMT-like process induced by MTDH in glioma cells

**Figure 6:**
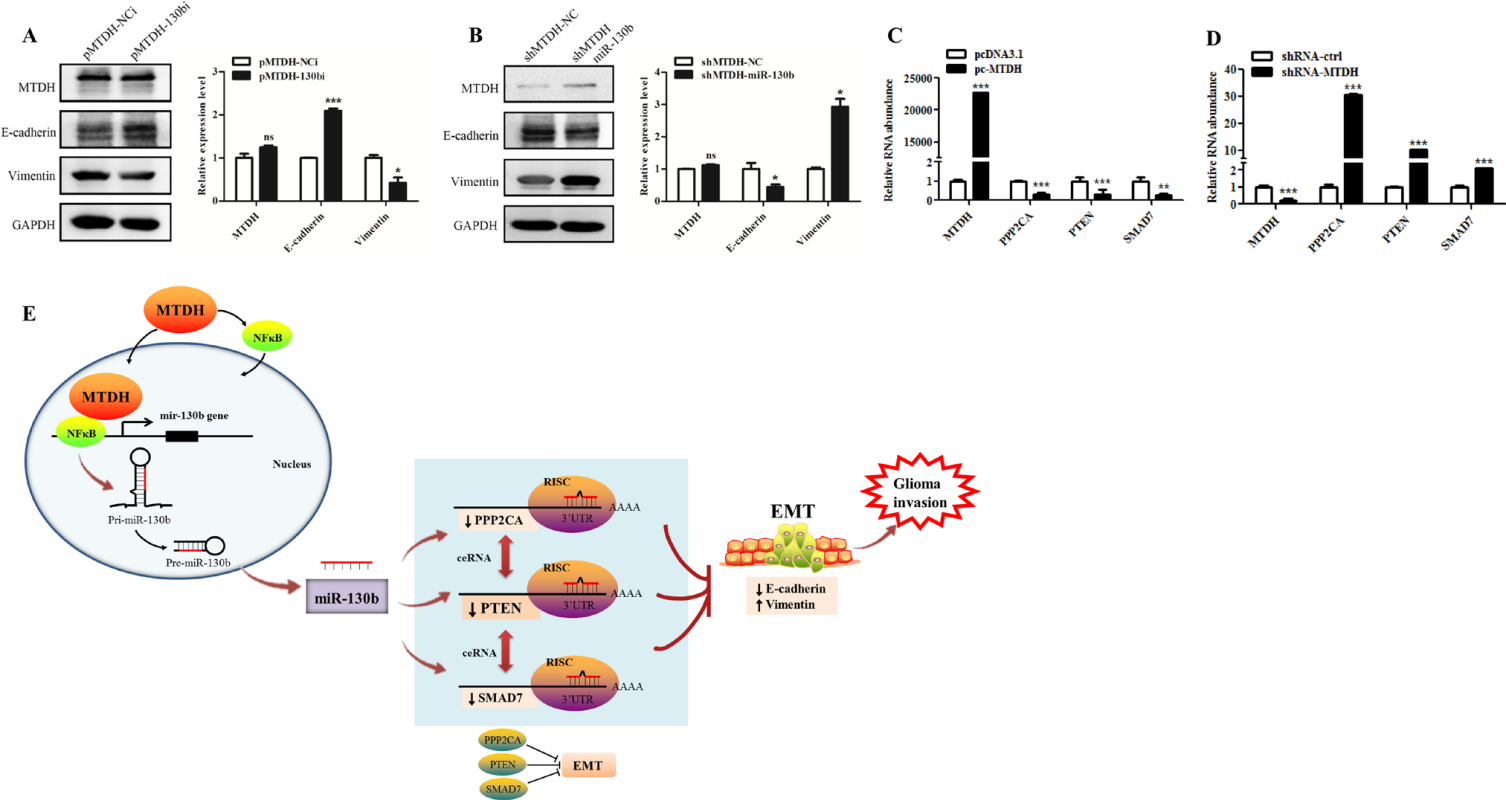
MiR-130b-ceRNAs mediates EMT-like process induced by MTDH in glioma cells (**A**) Western blot analysis of E-cadherin and Vimentin expression levels in MTDH overexpressing cells (pMTDH-U87) with miR-130b knockdown (or control). (**B**) Analysis of E-cadherin and Vimentin expression in MTDH knockdown cells (shMTDH-U87) with miR-130b overexpression (or control). ns, not significant, *p >* 0.05. (**C**, **D**) Quantitative RT-PCR analysis of the expression of miR-130b targets in glioma cells with MTDH overexpression or knockdown. Data represent mean ± SEM of three replicates. (**E**) Schematic model illustrating the mechanism by which MTDH drives glioma invasion. MTDH induces EMT-like process through modulating miR-130b-ceRNAs (PTEN/PPP2CA/SMAD7) to trigger glioma cell invasion. Red arrows denote potential interactions (our work), and black arrows denote validated interactions.

We further investigated the effect of miR-130b on EMT-like process regulated by MTDH. Stable MTDH overexpressing U87 cells were treated with miR-130b inhibitor (or control), while U87 cells with stable MTDH knockdown were transfected with miR-130b mimic (or control). Western blot analysis showed that knockdown of miR-130b in MTDH overexpressing U87 cells led to increased E-cadherin expression and decreased Vimentin expression (Figure 6A). While overexpression of miR-130b in MTDH knockdown U87 cells led to reduced E-cadherin expression but increased Vimentin expression (Figure 6B). These results suggest that miR-130b mediates EMT-like process induced by MTDH in glioma cells.

Furthermore, we found that MTDH overexpression restrained the expression levels of miR-130b ceRNAs (PTEN, PPP2CA and SMAD7) (Figure 6C). In contrast, MTDH knockdown enhanced PTEN, PPP2CA and SMAD7 expression levels (Figure 6D). Thus MTDH may regulate the expression of miR-130b-ceRNAs (PTEN/PPP2CA/SMAD7), which in turn regulate EMT-like process and glioma invasion (Figure 6E).

## DISCUSSION

MTDH has emerged as a crucial oncogene that participates in every aspect of cancer development and progression [3–5]. MTDH promotes glioma invasion via the upregulation of invasion related genes such as MMP2, MMP9 and claudin 4 [14, 15]. Here we demonstrate a novel mechanism of glioma invasion triggered by MTDH. MTDH promotes miR-130b expression and subsequently downregulates ceRNAs (PTEN, PPP2CA and SMAD7) to induce EMT-like process of glioma cells.

Based on analysis of TCGA database, we found that MTDH and mesenchymal markers are highly expressed in glioma, and MTDH expression level is positively correlated with mesenchymal marker Vimentin but negatively correlated with epithelial marker E-cadherin. In addition, glioma cells overexpressing MTDH exhibited enhanced mesenchymal characteristic and invasive capability, while glioma cells with MTDH depletion exhibited the reverse phenotypes. We further demonstrated that MTDH could modulate the expression of glioma-related miRNAs, especially miR-130b. Importantly, miR-130b overexpression promoted EMT-like process and invasion in glioma cells, whereas miR-130b downregulation inhibited EMT-like change and invasion in glioma cells. In addition, we identified PTEN as the miR-130b target that functions as the ceRNAs of PPP2CA and SMAD7 and exhibits inhibitory effects on glioma EMT-like process and invasion. Moreover, we provided evidence that the stimulatory effects of MTDH on EMT-like process and invasion of glioma cells are mediated by miR-130b.

MTDH has been implicated in EMT process of several tumor cells. In breast cancer cells, MTDH overexpression promoted cancer cells to undergo EMT, while MTDH knockdown reversed EMT changes [16]. In addition, MTDH could regulate TWIST expression in breast cancer cells [17]. MTDH promoted EMT of tongue squamous cell carcinoma (TSCC) through activating Wnt/PCP signaling pathway [6]. In non-small cell lung carcinoma (NSCLC), MTDH induced EMT through activating Wnt/beta-catenin signaling [18]. In other cancer cells, MTDH was found to induce EMT through the activation of p38 MAPK or AKT signaling. However, no studies have reported that MTDH regulates EMT in cancer cells via miRNA regulation network. In this study, for the first time we revealed that MTDH could induce EMT-like change of glioma cells through regulating miRNA expression.

Recent studies have documented the connection between MTDH and miRNAs, and most of them investigated miRNAs targeting MTDH [19, 20], but few studies investigate how MTDH regulates miRNAs. MTDH has been shown to activate NF-κB pathway and act as the cooperator for NF-κB to modulate downstream gene expression, such as IL8 [12, 21]. Transcription factor NF-κB can drive numerous miRNAs transcription in addition to the transcription of protein coding genes. For instance, NF-κB regulates miR-221/222, miR-125b, miR-9 and miR-155 expression [22–24]. These results are consistent with our data that MTDH modulates miR-130b expression depending on NF-κB subunit p65 activity. However, we could not exclude other mechanisms responsible for MTDH stimulated miR-130b expression, such as epigenetic mechanism. MTDH has been involved in epigenetic activation of TWIST1 [17]. In addition, numerous studies have indicated that epigenetic modifications such as aberrant DNA methylation and histone modification affect miRNA expression in cancer cells [25]. Therefore, the mechanism underlying MTDH regulated miRNA expression requires further investigation.

MiR-130b is known to play oncogenic role in various cancers, such as HCC, breast cancer and endometrial cancer [26, 27]. Overexpression of miR-130b enhances cancer stem cell-like phenotypes and increases the malignancy of cancer cells, such as proliferation, invasion and metastasis [25–29]. However, in pituitary adenoma, pancreatic cancer and thyroid carcinoma, miR-130b is downregulated and exerts reverse effects [30–32]. MiR-130b may act as an oncogenic molecule or tumor suppressor, depending on different targets and different cancer contexts. In our study, we found that miR-130b was overexpressed in glioma tissues and cells and enhanced glioma cell motility (Supplementary Figure S3), migration and invasion, indicating its oncogenic role in glioma. It has been reported that alteration of miR-130b expression regulated cell migration and invasion in glioma [33]. Additionally, previous reports have demonstrated that miR-130b regulated EMT through targeting PPARG in HCC, colorectal cancer and glioma and through targeting DICER1 in endometrial cancer [33–35]. In agreement with previous studies, here we showed that miR-130b induced EMT-like process of glioma cells by targeting PTEN.

CeRNA mechanism provides novel insight into the regulation of gene expression: ceRNAs communicate with and co-regulate each other by competing for binding to the shared microRNAs [36]. PTEN is a typical ceRNA for many miRNAs to regulate multiple RNA transcripts, affecting cancer development and progression. PTENP1, VAPA and CNOT6L could act as PTEN ceRNAs and regulate PTEN expression [36]. PTEN downregulation led to the induction of EMT, decreased cell adhesion and increased cell motility and invasiveness in various cancer cells [37–39]. In this study, we identified novel PTEN-associated ceRNAs, PPP2CA and SMAD7. PPP2CA could reverse EMT and suppress prostate cancer growth and metastasis [40]. SMAD7 is a TGF-β inhibitor and inhibits EMT process in various cancer cells [41]. Based on these data, we speculated that PTEN might exert more powerful regulatory effects on EMT through mediating ceRNA cross-talk in addition to through the regulation of PI3K/AKT pathway.

In conclusion, we demonstrate that in glioma cells MTDH acts as a co-activator for NF-kB to upregulate miR-130b expression, which in turn suppresses PTEN, PPP2CA and SMAD7 expression. Consequently, glioma cells undergo EMT-like process and exhibit enhanced invasion. These findings reveal a new mechanism by which MTDH drives glioma invasion. To the best of our knowledge, this is the first report that MTDH could regulate miRNA expression, which broadens our understanding of MTDH function. Our data suggest that MTDH may have great potential in controlling enormous miRNA regulatory networks to regulate tumorigenesis, and further confirm that MTDH is a promising target for cancer prevention and therapy.

## MATERIALS AND METHODS

### Database and bioinformatics analysis

All mRNA and miRNA expression datasets for GBM and normal brain were obtained from TCGA database (http://cancergenome.nih.gov/), and Perl and R scripts were used to analyze the normalized level 3 data. Epithelial and mesenchymal genes and EMT-related genes were collected from Gene ontology dataset (http://geneontology.org/), Gene Set Enrichment Analysis (GSEA) plate and literatures. Unsupervised hierarchical clustering was conducted to analyze the expression of epithelial and mesenchymal markers in normal brain and glioma. Correlation analyses were performed to analyze the relationship between MTDH and EMT markers (E-cadherin and Vimentin) and the relationship among EMT-related genes in glioma samples from TCGA. MiRNA targets were identified by using predicted and validated miRNA-target interactions software programs, including Targetscan, RNAhybrid, RNA22, Pictar, miRTarbase and miRwalk. Transcription factor prediction was conducted based on ChIPBase database (http://rna.sysu.edu.cn/chipbase/) and the ChIP-seq data analyses were visualized using UCSC genome viewer (http://genome.ucsc.edu/).

### Plasmids, small interfering RNA (siRNA), miRNA mimic and inhibitor

MTDH expression plasmid pcDNA3.1-MTDH (pc-MTDH) and control plasmid pcDNA3.1 were kindly provided by Prof. Mengfeng Li (Sun Yat-sen University, Guangzhou, China). Pwslv-sh02-MTDH plasmid (shRNA-MTDH) was purchased from VlewSolid Biotech (Beijing, China). PRS-sh4-MTDH (sh4-MTDH) plasmid and its control PRS-shRNA (shRNA) were kindly provided by Prof. Xiangbing Meng (University of Iowa, IA, USA). SiRNA-p65 (sip65), miR-130b mimic, miR-130b inhibitor and their negative controls were purchased from GenePharma (Shanghai, China). sip65 was designed to target NF-κB p65 subunit as described previously [5]. The sequences of miRNA mimic and inhibitor were as follow: has-miR-130b mimic (sense, 5′-CAGUGCAAUGAUGAA AGGGCAU-3′, antisense, 5′-UUGUCACGUUACUACU UUCCC G-3′), and has-miR-130b inhibitor (5′-AUGC CCUUUCAUCAUUGCACUG-3′). Lentivirus packaging plasmid DR8.9 and envelope plasmid VSVG were kindly provided by Prof. Haisheng Zhou (Anhui Medical University, Hefei, China). PTEN overexpression plasmid (pc-PTEN) was kindly provided by Prof. Yan Jin (Harbin Medical University, Harbin, China)

### Cell culture, transfection, and lentivirus

Human glioma cell lines (U87, U251, T98G, SHG44, LN229) and HEK293T cell line were purchased from the Chinese Academy of Sciences (Shanghai, China). All cell lines were cultured in DMEM supplemented with 10% fetal bovine serum (Invitrogen, Carlsbad, CA, USA) and 1% penicillin-streptomycin (Invitrogen, USA). Glioma cells were transiently transfected with plasmids, siRNAs or miRNA mimic and inhibitor using Lipofectamine 2000 (Invitrogen, USA). Stable U87 cells with the overexpression of MTDH were selected for 2 weeks in 0.8 mg/mL G418 (Amresco, Solon, OH, USA). U87 cells with steadily knockdown of MTDH (sh4-MTDH) were selected for 2 weeks in 1 μg/mL puromycin (Sigma, Aldrich Deisenhofen, Germany). Lentivirus for shRNA-MTDH were generated in HEK-293T using pwslv-sh02-MTDH and packing vectors (DR8.9 and VSVG), and then the lentivirus was used to infect U87 cells in 0.5% polybrene (Sigma Aldrich Deisenhofen, Germany).

### MTT assay

U87 cells with MTDH overexpression or knockdown were seeded in 96-well plates. At 24 h, 48 h and 72 h, 10 μL MTT solution (5 mg/mL, Sigma, Germany) was added and the cells were incubated for 4 h. Subsequently, cell medium was removed and 150 μL DMSO (Amresco, USA) was added into each well. After formazan crystals were dissolved, OD measurement was performed at 490 nm by microplate reader (Bio-Rad, USA). Cell viability was measured based on absorption value.

### Wound healing assay

Stable U87 cells with MTDH overexpression or knockdown were seeded in 6-well plates and cultured until confluency. A scratched wound was made using a sterile pipette tip. The wounded monolayers were washed twice with PBS to remove non adherent cells and incubated in DMEM without FBS. Photographs of wound healing were taken at 0 h and 24 h after scratching under an Axiovert 200 microscope (Carl Zeiss, Germany) and measured by Image pro-plus software. The experiments were performed in triplicate.

### Transwell invasion assay

The transwell invasion assay was performed in 24-well cell culture chambers using transwell inserts (Corning, USA) with 8-μm pore size. The membranes of transwell inserts were coated with Matrigel (BD Bioscience, USA) diluted in DMEM at the ratio of 1:5. Briefly, 5 × 10^4^ transfected cells were resuspended in 100 μL serum-free medium and plated into upper chamber. In the lower chamber, DMEM supplemented with 10% FBS (600 μL /well) was added as chemoattractant. After the cells were incubated for 24 h, upper surface of the insets were removed with a cotton swab, while lower surface were fixed with methanol for 30 min, air dried and stained with 0.1% crystal violet solution. The invading cells were viewed and counted under microscope. All experiments were carried out in triplicate.

### Real-time PCR

Total RNA was extracted from cultured cells using Trizol reagent (Invitrogen, USA). The cDNAs were synthesized from total RNA with the PrimeScript RT reagent kit (Takara, Japan) following the manufacturer's instruction. Real-time PCR was performed using SYBR Premix Ex Taq (Takara, Japan) in triplicate. Primers synthesized by Sangon Biotech (Shanghai, China) were as follows: *miR-130b*, 5′-GTC GTA TCC AGT GCA GGG TCC GAG GTA TTC GCA CTG GAT ACG ACA TGC CCT-3′ (RT) , 5′-GCG GCG GCA GTG CAA TGA TGA AAG-3′ (forward) and 5′-ATC CAG TGC AGG GTC CGA GG-3′ (reverse); *U6*, 5′-CGC TTC ACG AAT TTG CGT GTC AT-3′ (RT), 5′-GCT TCG GCA GCA CAT ATA CTA AAAT-3′ (forward) and 5′-CGC TTC ACG AAT TTG CGT GTC AT-3′ (reverse); *MTDH*, 5′-AAA TAG CCA GCC TAT CAA GAC TC-3′ (forward) and 5′-TTC AGA CTT GGT CTG TGA AGG AG-3′ (reverse); *E-cadherin*, 5′-TTG CTA CTG GAA CAG GGA CAC-3′ (forward) and GGA GAT GTA TTG GGA GGA AGG-3′ (reverse); *Vimentin*, 5′-CAG GCT CAG ATT CAG GAA CAG-3′ (forward) and 5′-GCA GCC ACA CTT TCA TAT TGC-3′ (reverse); *p65*, 5′-GGG AAG GAA CGC TGT CAG AG-3′ (forward) and 5′-TAG CCT CAG GGT ACT CCA TCA-3′ (reverse); *PPP2CA*, 5′-CGC CTA CAA GAA GTT CCC CA-3′ (forward) and 5′-TCT AGA CAC CAA CGT GAG GC-3′ (reverse); *PTEN*, 5′-AGA CAT TAT GAC ACC GCC AAA-3′ (forward) and 5′-AAG TTC TAG CTG TGG TGG GTT-3′ (reverse); *GAPDH*, 5′-ATC ACT GCC CAC CCA GAA GAC-3′ (forward) and 5′-TTT CTA GAC GGC AGG TCA GG-3′ (reverse). MiRNA levels were normalized to *U6*, while mRNAs levels were normalized to *GAPDH*. The relative quantitation values for miRNA and mRNA expression were calculated by the 2^–ΔΔct^ method.

### Western blot analysis

Total protein was isolated from glioma cells using cell lysis buffer (Beyotime, Shanghai, China), separated by SDS–PAGE, and transferred to PVDF membranes. Membranes were incubated with primary antibodies for MTDH, E-cadherin (rabbit polyclonal, Santa Cruz, USA), Vimentin (mouse monoclonal, Boster, Wuhan, China), PTEN (mouse monoclonal, Proteintech Group Inc, Chicago, USA) and GAPDH (ZSGB-Bio, Beijing, China), followed by incubation with HRP conjugated secondary antibodies and ECL detection. GAPDH was used as loading control. All experiments were performed in triplicate.

### Statistical analysis

Statistical analyses were performed using the SPSS version 13 package (SPSS Inc., Chicago, IL, USA). The difference between two groups was assessed by independent Student's *t-test* and Chi-square test. Pearson correlation analysis was conducted to analyze the correlation. *p* < 0.05 was considered statistically significant.

## SUPPLEMENTARY MATERIALS FIGURES AND TABLES


