# Supplementary material for: MTDH promotes glioma invasion through regulating miR-130b-ceRNAs
Source: Oncotarget. 2017 Jan 18;8(11):17738–49. doi: 10.18632/oncotarget.14717 (PMC5392282; doi:10.18632/oncotarget.14717)
Supplement: Supplementary file 1 [file oncotarget-08-17738-s001.pdf]

MTDH promotes glioma invasion through regulating miR-130b-ceRNAs

Supplementary Materials

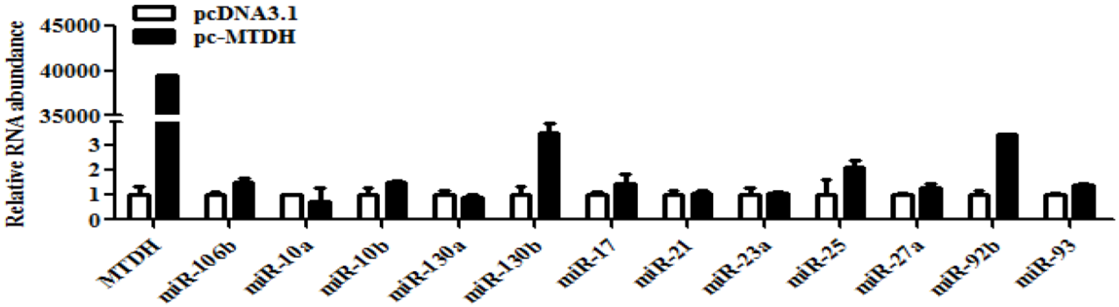

Supplementary Figure S1: MTDH overexpression changes the expression of glioma-related miRNAs. Quantitative RT-PCR analysis of the expression changes of miRNAs in glioma cells with MTDH overexpression.

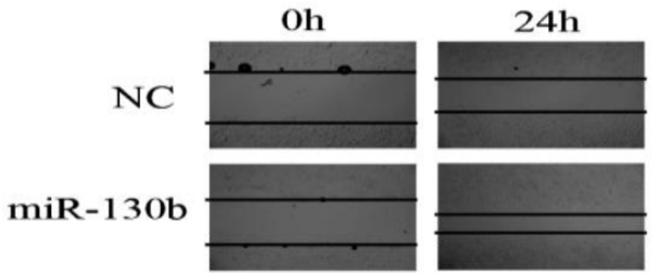

Supplementary Figure S2: MiR-130b promotes glioma cells migration. Cell migration was assessed by wound-healing assay in glioma cells treated with miR-130b mimic or control mimic. The wound was photographed at 0 h and 24 h after scratching.

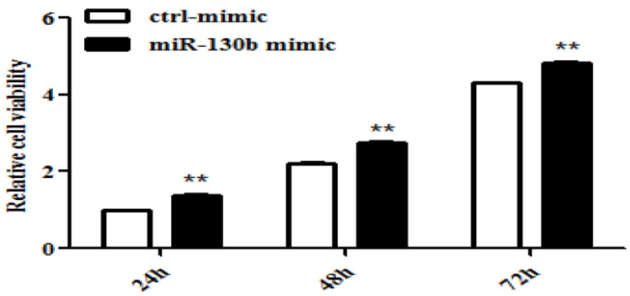

Supplementary Figure S3: MiR-130b promotes glioma cells viability. Cell viability was analyzed by MTT assay in different time intervals after transfection with miR-130b mimic or control mimic.

Supplementary Table S1: Important miRNAs in glioma

MiRNA name	Glioma (papers)	Array studies (upregulation)
miR-106b	7	6
miR-10a	3	4
miR-10b	11	8
miR-130a	1	6
miR-130b	2	5
miR-155	9	8
miR-17	3	2
miR-182	6	2
miR-21	61	18
miR-210	3	5
miR-221	22	6
miR-222	19	5
miR-23a	5	7
miR-25	2	5
miR-27a	4	2
miR-92b	2	3
miR-93	2	3

Supplementary Table S2: Prediction of EMT-related genes targeted by miR-130b

miRNA Name	Gene Name	target Scan Sites	picTar Sites	RNA22 Sites	PITA Sites	miRanda Sites	Cancer Num
hsa-miR-130b-3p	DICER1	2 [14,1398]	6 [25, 1937]	1 [11, 515]	2 [14, 1398]	2 [14, 1398]	3
hsa-miR-130b-3p	FOXF2	1 [12, 1294]	1 [12, 1294]	0 [0, 0]	1 [12, 1294]	1 [12, 1309]	5
hsa-miR-130b-3p	PPARG	1 [4, 67]	1 [4, 67]	1 [4, 67]	1 [4,67]	1 [4, 67]	8
hsa-miR-130b-3p	PPP2CA	0 [0, 0]	0 [0, 0]	0 [0, 0]	0 [0,0]	1 [8, 328]	1
hsa-miR-130b-3p	PTEN	2 [9, 694]	6 [21,1859]	0 [0, 0]	2 [9, 694]	2 [10, 695]	9
hsa-miR-130b-3p	SMAD7	0 [0, 0]	0 [0, 0]	1 [10, 1695]	0 [0, 0]	1 [10, 1686]	8
hsa-miR-130b-3p	TGFBR3	0 [0, 0]	0 [0, 0]	0 [0, 0]	0 [0, 0]	1 [1, 304]	8
hsa-miR-130b-3p	TIMP2	1 [3, 2936]	2 [3, 2937]	0 [0, 0]	1 [3, 2936]	1 [4, 2940]	8
hsa-miR-130b-3p	TRPS1	1 [4, 59]	6 [7, 364]	0 [0, 0]	1 [4, 59]	1 [4, 59]	5

Supplementary Table S3: Correlation of EMT-related genes co-expression in glioma based on TCGA database

Coefficient	DICER1	FOXF2	PPARG	PPP2CA	PTEN	SMAD7	TGFBR3	TIMP2	TRPS1
DICER1	1	0.334824	–0.131554	0.152629	0.292250	0.362836	0.291900	0.098598	0.451826
FOXF2	0.334824	1	0.047417	0.219764	0.347695	0.238016	0.099759	0.094575	0.263704
PPARG	–0.131554	0.047417	1	–0.063591	0.204239	0.279010	0.062529	0.193907	–0.257958
PPP2CA	0.152629	0.219764	–0.063591	1	0.320249	0.155310	–0.108465	0.260475	0.186730
PTEN	0.292250	0.347695	0.204239	0.320249	1	0.421080	0.127343	0.296744	0.134502
SMAD7	0.362836	0.238016	0.279010	0.155310	0.421080	1	0.275479	0.139450	0.218336
TGFBR3	0.291900	0.099759	0.062529	–0.108465	0.127343	0.275479	1	–0.025703	0.419050
TIMP2	0.098598	0.094575	0.193907	0.260475	0.296744	0.139450	–0.025703	1	0.122319
TRPS1	0.451826	0.263704	–0.257958	0.186730	0.134502	0.218336	0.419050	0.122319	1
